# Supplementary material for: Emotion dynamics in movie dialogues
Source: PLoS One. 2021 Sep 20;16(9):e0256153. doi: 10.1371/journal.pone.0256153 (PMC8452047; doi:10.1371/journal.pone.0256153)
Supplement: Attachment — Submitted filename: response.docx [file pone.0256153.s001.docx]

**Official Response Letter**

Dear editor and reviewers. Thank you for your thoughtful comments. We have made several changes to address them. Details are below (Reviewer comments are italicized):

Reviewer 1 comments:

*1. I think it would be better to move data description part in front of method section. So the readers would have a clear picture about how the values are generated.*

Because the metrics can be applied to a variety of data sources, we opted to introduce the metrics first followed by the description of the movie scripts data as the application of the metrics. However, we have included a statement at the beginning of the method (Section: Utterance Emotion Dynamics) explaining that UED metrics can be extracted from a variety of data sources. We also include a pointer to the section specifically describing the Movie Dialogues data.

*2. Applied methods should be written in one section, for example, the hypothesis testing.*

Because of the novelty of the UED metrics, we structured the paper in a way to build complexity and show how the UED metrics can be applied to answer a variety of interesting questions. The first set of results focus on how we can use UED to describe characters’ emotion dynamics. The second set of results focuses squarely on testing hypotheses about wider trends across stories and character interactions. We think this structure helps the reader understand how to use the UED metrics to answer increasingly complex questions about emotion dynamics.

*2.  Table captions should be at the top of the tables.*

Fixed.

*3. Lines 24 and 25 are not needed.*

These lines are now a footnote.

*4. in Table 2, please define what is "displacement count".*

We have added a sentence in the UED metrics section more clearly defining displacement count and length.

*5. Figure 4, please explain what is "the character's location in the v-a space".*

Done. We added a sentence describing that it is the character’s v-a rolling average when the word was uttered.

*6. Line 261, typo: "be explore"?*

Fixed.

*7. Figures 1 and 2, the presented two characters are chosen randomly? Please explained.*

Done. We clarify that the two characters chosen were two main characters.

*8. Figure 6, it would be better to add S.D. or 1-, 3-quartile in addition to average, to display the variations.*

Done. We added 95% confidence bands.

*9. About the character-character discordances, is this done by all possible pairs or selected? Please clarify.*

We added a sentence clarifying that discordances were calculated for all possible pairs of characters (who met the inclusion criteria) within each movie.

*10. Lines 187-188, "a turn as a sequence of uninterrupted utterances by a character". What is "uninterrupted", a character continues to talk, without stops? Do scenes change count as interruption?*

We added the following sentence to make this clearer: In other words, a turn begins when a character’s dialogue begins, and ends when either a different character’s dialogue starts or the movie ends. Scene changes do not count as an interruption if the same character speaks at the end of one scene and at the start of the next scene. (This is okay here because we continue to track the emotional state of the character through their utterances before and after scene change.)

*11. Result section should be made more clear.*

We made the following changes to improve the results section:

1. We reduced the amount of reiteration of related work and interpretation that is covered in earlier or later sections. The idea is to keep the results section more clearly focused on the actual findings, while leaving the bulk of contextual information for other sections.
2. We streamlined the reporting of statistical results. We omit overt references to the null hypotheses as these are often clear from the context of the statistical test.
3. Some tangential statistical tests have been made into footnotes. For example, in the hypothesis test of peak discordance, we save the test conducted on the subset of characters present at the beginning and end 10% of the narrative for a later footnote.

Reviewer #2 comments:

*Great subject, which might attract specific interest in various sciences such as neuroscience, psychology, forensic and psychotherapy and performing arts in addition to the relevant industries. Considering recent pandemic which has promoted various meeting via visual technologies, this piece of research would be warmly greeted. It would be interesting to see the further similar studies by the authors and other interested scientists.*

Thank you for the support and encouragement.  We have included some connections of this work, especially with psychology and public health, but we welcome suggestions on specific ways to lnk this work to the various fields you have mentioned.
